# Regulation of Budding Yeast Mating-Type Switching Donor Preference by the FHA Domain of Fkh1

**DOI:** 10.1371/journal.pgen.1002630

**Published:** 2012-04-05

**Authors:** Jin Li, Eric Coïc, Kihoon Lee, Cheng-Sheng Lee, Jung-Ae Kim, Qiuqin Wu, James E. Haber

**Affiliations:** Department of Biology and Rosenstiel Center, Brandeis University, Waltham, Massachusetts, United States of America; University of California Davis, United States of America

## Abstract

During *Saccharomyces cerevisiae* mating-type switching, an HO endonuclease-induced double-strand break (DSB) at *MAT* is repaired by recombining with one of two donors, *HML*α or *HMR* **a**, located at opposite ends of chromosome III. *MAT* **a** cells preferentially recombine with *HML*α; this decision depends on the Recombination Enhancer (RE), located about 17 kb to the right of *HML*. In *MAT*α cells, *HML* is rarely used and RE is bound by the MATα2-Mcm1 corepressor, which prevents the binding of other proteins to RE. In contrast, in *MAT* **a** cells, RE is bound by multiple copies of Fkh1 and a single copy of Swi4/Swi6. We report here that, when RE is replaced with four LexA operators in *MAT* **a** cells, 95% of cells use *HMR* for repair, but expression of a LexA-Fkh1 fusion protein strongly increases *HML* usage. A LexA-Fkh1 truncation, containing only Fkh1's phosphothreonine-binding FHA domain, restores *HML* usage to 90%. A LexA-FHA-R80A mutant lacking phosphothreonine binding fails to increase *HML* usage. The LexA-FHA fusion protein associates with chromatin in a 10-kb interval surrounding the HO cleavage site at *MAT*, but only after DSB induction. This association occurs even in a donorless strain lacking *HML*. We propose that the FHA domain of Fkh1 regulates donor preference by physically interacting with phosphorylated threonine residues created on proteins bound near the DSB, thus positioning *HML* close to the DSB at *MAT*. Donor preference is independent of Mec1/ATR and Tel1/ATM checkpoint protein kinases but partially depends on casein kinase II. RE stimulates the strand invasion step of interchromosomal recombination even for non-*MAT* sequences. We also find that when RE binds to the region near the DSB at *MAT* **a** then Mec1 and Tel1 checkpoint kinases are not only able to phosphorylate histone H2A (γ-H2AX) around the DSB but can also promote γ-H2AX spreading around the RE region.

## Author Summary

Mating-type gene switching occurs by a DSB–initiated gene conversion event using one of two donors, *HML* or *HMR*. *MAT* **a** cells preferentially recombine with *HML* whereas *MAT*α cells choose *HMR*. Donor preference is governed by the Recombination Enhancer (RE), located about 17 kb from *HML*. RE is repressed in *MAT*α cells, whereas in *MAT* **a** RE binds several copies of the Fkh1 protein. We replaced RE with four LexA operators and showed that the expression of LexA-Fkh1 fusion protein enhances *HML* usage. Donor preference depends on the phosphothreonine-binding FHA domain of Fkh1. LexA-FHA_Fkh1_ physically associates with chromatin in the region surrounding the DSB at *MAT*. We propose that RE regulates donor preference by the binding of FHA_Fkh1_ domains to phosphorylated sites around the DSB at *MAT*, thus bringing *HML* much closer than *HMR*. FHA_Fkh1_ action partially depends on casein kinase II but not on the DNA damage checkpoint kinases Mec1 and Tel1. We also find that, when RE binds to the *MAT* region, phosphorylation of histone H2A (γ-H2AX) by Mec1/Tel1 not only surrounds the DSB but also spreads around RE. This is the first demonstration that γ-H2AX can spread to contiguous, but undamaged, chromatin.

## Introduction

**Figure 1:**
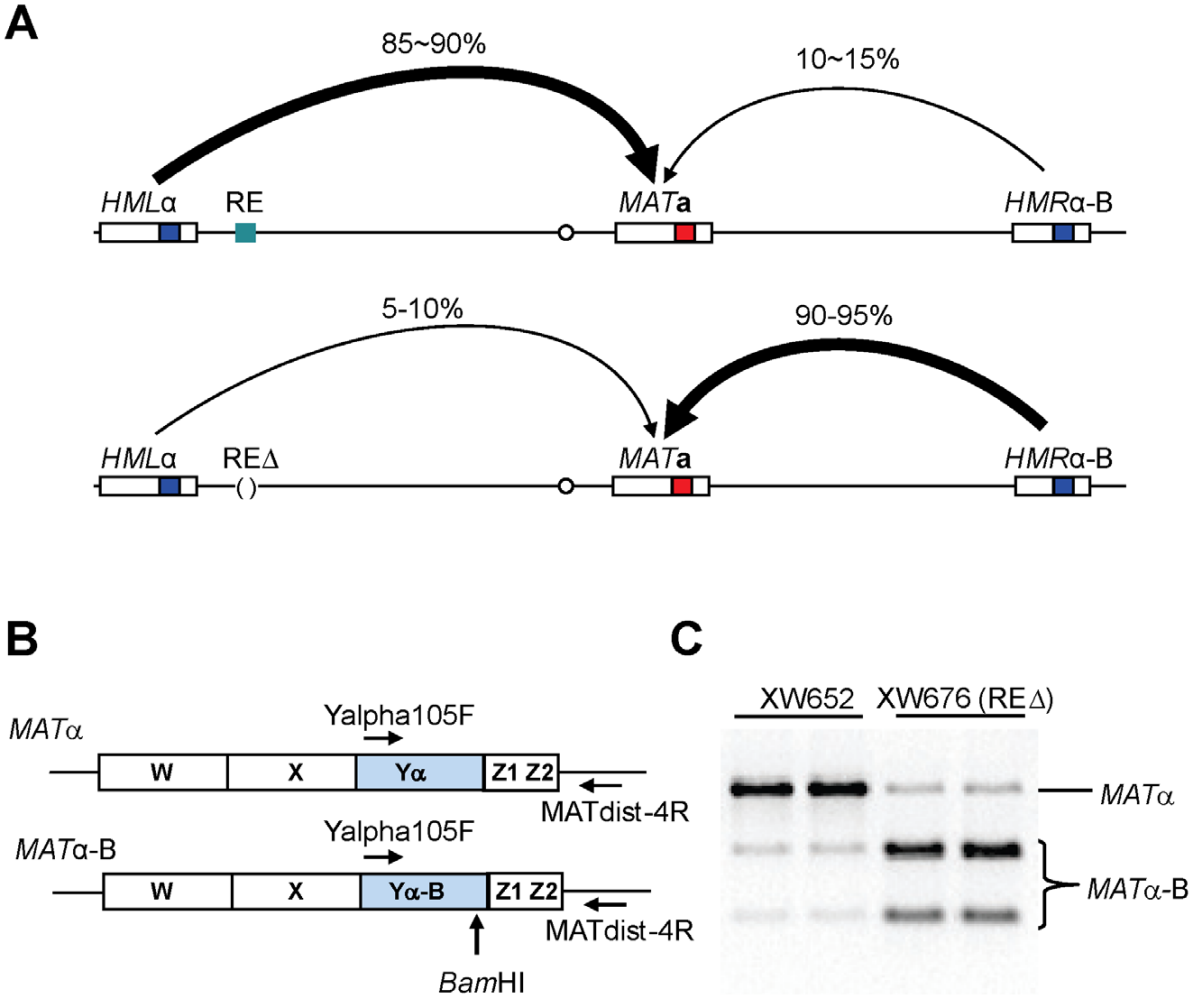
Measure Donor Preference via a PCR-Based Assay. (A) Mating-type switch at the *MAT* locus. When RE is active in *MAT* **a** cells, donor preference (*HML* usage) is 85∼90%. In contrast, *HML* usage reduces to only 10∼15% when RE is deleted. Donor preference is calculated using a formula (*MATα*/(*MATα+MATα-*B)). (B) A PCR-based strategy for measuring donor preference. Diagrams are shown for *MAT*α and *MAT*α-B. After galactose induction, DSBs at *MAT* can be repaired using either donor of *HML*α and *HMR*α*-*B. A primer pair (Yalpha105F/MATdist-4R) can only amplify *MAT*α or *MAT*α-B, but not *HML*α, *HMR*α-B, *MAT* **a** due to specificities of these two primers. (C) Measure donor preference via a PCR-based assay. Both *MAT*α and *MATα-*B are PCR-amplified using the primer pair (Yalpha105F/MATdist-4R). The purified PCR products are digested with *BamH*I and checked on ethidium bromide stained agarose gel. RE is present in XW652, but deleted in XW676.

*Saccharomyces* mating-type switching occurs through a DSB-initiated intrachromosomal gene conversion event at *MAT*, using one of two donors on chromosome III, *HML* and *HMR* (Figure 1A) [1]–[3]. Switching is initiated by expression of the site-specific HO endonuclease that cleaves only one site in the yeast genome, *MAT* **a** or *MAT*α. The unexpressed mating-type genes in *HML*α and *HMR* **a** also contain HO cleavage sites, but they are not cut because these regions are heterochromatic [4]–[6]. Although either *HML*α or *HMR* **a** can be used to repair a DSB at *MAT*, there is a strong mating type-dependent preference for the choice of the two donors. In *MAT* **a** cells, *HML*α is preferentially chosen for repair, about 85–90% of the time, whereas *MAT*α cells strongly prefer *HMR* **a**, about 95% [3], [7]–[9]. Donor preference is not altered if the mating-type genes encoded in the Y region are changed, e.g. if *HMR* carries Yα instead of Y**a** or if *HML* is replaced with *HMR* [7], [8].

Donor preference in *MAT* **a** depends on an approximately 275-bp Recombination Enhancer (RE), located 17 kb to the right of *HML* [10], [11]. One important aspect of donor preference is that *MAT* **a** cells activate a large (∼40 kb) region near the left end of chromosome III, so that a donor within this region is strongly preferred over *HMR* [8]. RE is responsible for this activation along the entire left arm of chromosome III [11], [12]. Donor preference does not change if the cis-acting silencer sequences around *HML* or *HMR* are removed [13]. In addition, RE is not limited to the special features of *MAT* switching. If a *leu2* allele is inserted in place of *HML*, its success in recombining with a different *leu2* allele, either near *MAT* or even on another chromosome, is 20–30 times higher in *MAT* **a** than in *MAT*α and is RE-dependent [8], [12].

RE is “portable;” that is, it will work in other chromosome contexts. When *HML*, *HMR* and *MAT* **a** are all inserted on chromosome V, *HML* usage increases significantly when RE is inserted nearby [12]. In addition, in *MAT* **a** cells where RE promotes *HML*, the usage of *HMR* can be markedly increased by placing a second RE near *HMR* [11], [12].

In *MAT*α cells, RE is inactivated by binding of the Matα2-Mcm1 repressor complex, which leads to formation of highly organized nucleosomes covering the RE region but not extending into adjacent gene regions [8], [10], [14]. In *MAT* **a** cells, RE exhibits several nuclease hypersensitive sites when Mcm1 binds RE in the absence of the Matα2 protein (which is not expressed in *MAT* **a** cells). In addition to the Matα2-Mcm1 operator region, RE is composed of several evolutionarily conserved chromatin domains [14], several of which were shown to contain putative binding sites for the Fkh1 transcription factor [15]. A conserved SCB (Swi4/Swi6 cell cycle box) is also present in Region C of RE [16]. Both Fkh1 and Swi4/Swi6 regulate donor preference by binding to RE in *MAT* **a** cells [15]–[17]. Despite the presence of these transcription factors, there are no open reading frames adjacent to RE, although there is an adjacent noncoding RNA [18]. The DNA repair proteins Ku70 and Ku80 have a small effect on *MAT* **a** donor preference that may be caused by the role of these proteins in localizing *HML* to the nuclear periphery [19]. Deleting the Chl1 helicase also causes a small reduction of *MAT* **a** donor preference without affecting *MAT*α choice [16], [20].

Despite the identification of several proteins that bind to RE, it is still not clear how RE regulates donor preference. Previously we showed that RE could be deleted and replaced with small modules derived from RE. Notably 4 tandem copies of a 22-bp sequence containing a putative Fkh1 binding site were sufficient to increase *HML* usage to >60% (where the use of *HML* in REΔ is 5%); this increased preference for *HML* is abolished in *fkh1*Δ [15]. To further explore the mechanism of RE regulation, we replaced RE with four LexA operators and found that a LexA-Fkh1 fusion strongly promotes *HML* usage. Using this system, we dissect Fkh1 and find out that RE activity depends on the phosphothreonine binding motif of the FHA domain of Fkh1 and not on its forkhead domain. We show that LexA-FHA_Fkh1_ becomes associated with the chromatin surrounding the *MAT* only after DSB induction. This interaction is seen even in a donorless strain, demonstrating that the FHA-mediated regulation is a break-dependent but repair-independent process. *MAT* **a** donor preference is partially dependent on casein kinase II but not on two checkpoint kinases, Mec1 and Tel1. We propose that the FHA_Fkh1_ domain regulates donor preference by physically interacting with phosphorylated threonines on histones or other bound proteins surrounding the DSB during mating-type switch.

## Results

### Measuring Donor Preference by Southern Blot or a PCR-Based Assay

All strains in this study are derived from XW652 [11], which carries *HML*α, *MAT* **a** and *HMR*α-B on chromosome III (Figure 1A). *HMR*α-B contains a single base pair change that creates a *BamH*I site [8]. After galactose-induced expression of *HO*, *MAT* **a** can be repaired to *MAT*α or *MAT*α-B, using *HML*α or *HMR*α-B, respectively. Following HO induction for 60 min, HO expression was repressed by the addition of 2% dextrose and the ratio of switching to *MAT*α or *MAT*α-B was checked after 24 h. Donor preference could be measured either by Southern blot [8] or by a PCR-based assay in which the combination of *MAT*α or *MAT*α-B PCR products is digested with *BamH*I (Figure 1B). PCR-based assay showed 85% usage of *HML*α for XW652 but ≤10% for RE-deleted XW676 (Figure 1C).

### A LexA System to Study the Regulation of Donor Preference

**Figure 2:**
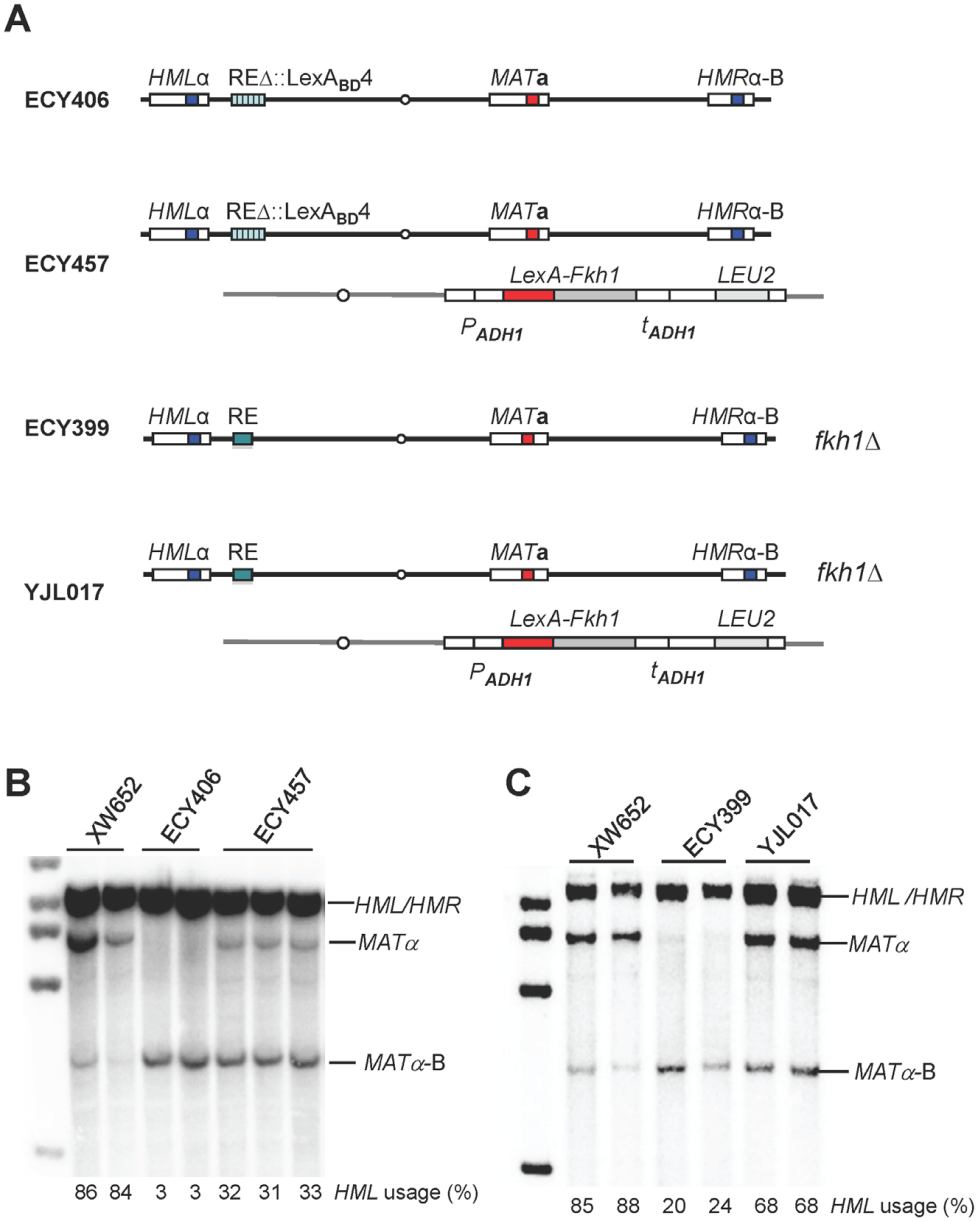
A LexA System to Study Donor Preference. (A) Illustration of strain genotypes for ECY406, ECY457, ECY399 and YJL017. (B) LexA-Fkh1 regulates donor preference by binding to REΔ::LexA_BD_4. ECY457 was constructed by integrating LexA-Fkh1 (from pEC16) to *arg5,6* of ECY406. Donor preference was measured by Southern blot using a Yα specific probe in panels B and C. XW652 serves as a wild-type control. (C) LexA-Fkh1 complements a *fkh1Δ* mutant (ECY399) to regulate donor preference presumably by binding to RE. The *arg5,6::LexA-Fkh1* was crossed into ECY399 to generate a strain YJL017.

Fkh1 is involved in the regulation of donor preference through direct interaction with RE [15], [16]. To further explore the role of Fkh1, we constructed a strain ECY406 by replacing RE with four LexA operators (Figure 2A). In an otherwise wild type background, *HML* usage in ECY406 was less than 5% as expected for a deletion of RE (Figure 2B). We then constructed a plasmid pEC16 that constitutively expresses a LexA-Fkh1 fusion protein from an *ADH1* promoter of pAT4 [21]. The LexA-Fkh1 sequences from pEC16 were stably integrated at the *arg5,6* locus of ECY406 to generate a new strain ECY457 (Figure 2A). Expression of LexA-Fkh1 in ECY457 was able to up-regulate donor preference to around 32% presumably by binding to four LexA operators replacing RE (Figure 2B), whereas the use of *HML* was less than 5% when LexA alone was expressed (data not shown). This result demonstrates that regulation of donor preference by Fkh1 does not require the binding of Mcm1 or Swi4/Swi6 to their specific sites in the normal RE sequences. We noted further that the Fkh1 moiety in the LexA-Fkh1 fusion remained functional even with normal RE, as it could complement a *fkh1*Δ mutant in YJL017 by up-regulating donor preference to 68% (Figure 2C).

### The Phosphothreonine-Binding FHA Domain of Fkh1 Is Responsible for Donor Preference

**Figure 3:**
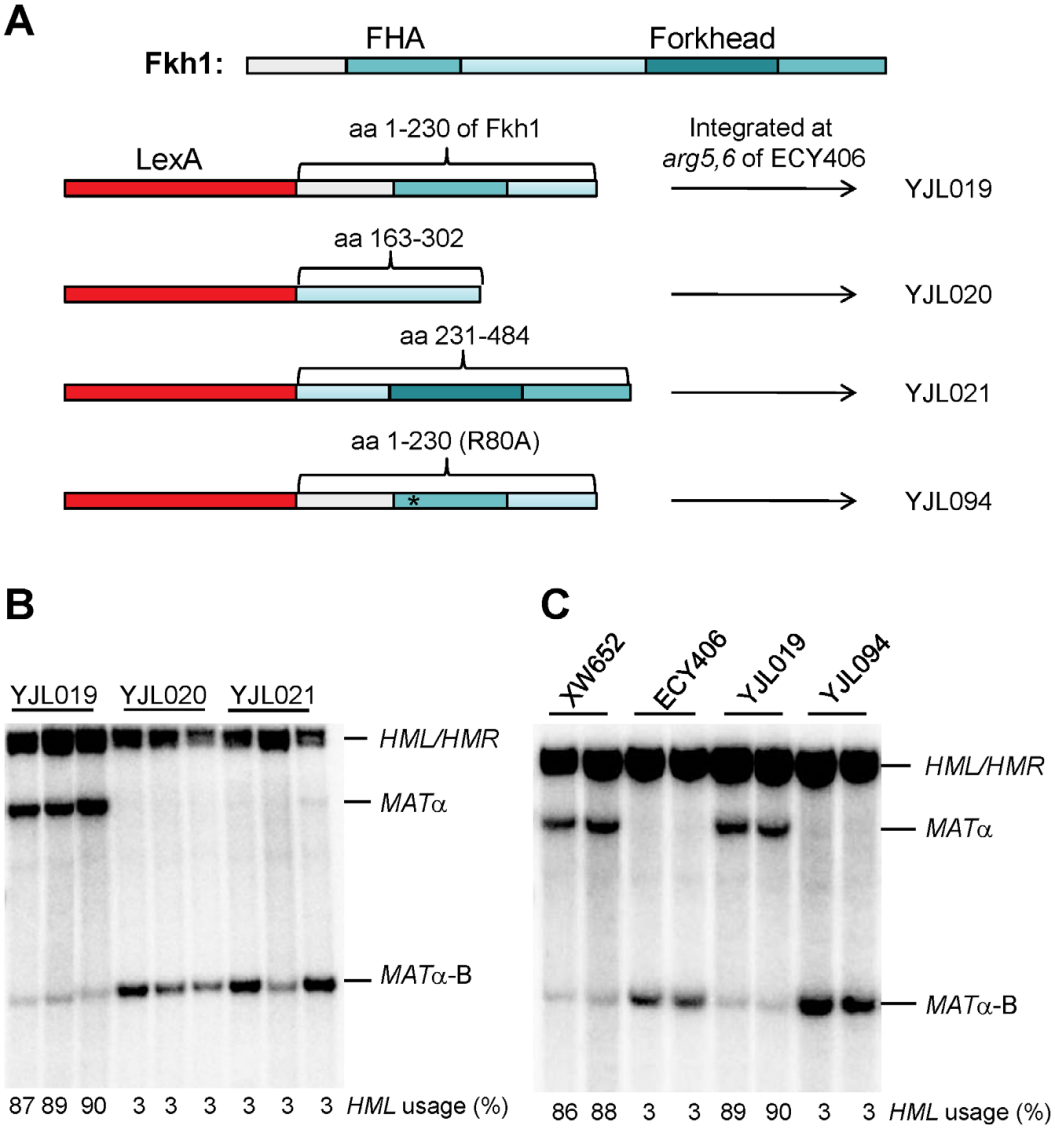
The FHA Domain of Fkh1 Is Responsible for Donor Preference Regulation. (A) The strain construction strategy for YJL019, YJL020, YJL021 and YJL094. Fkh1 has two conserved domains: FHA and a forkhead DNA binding domain. We prepared three plasmid constructs by fusing LexA of pAT4 with different regions of Fkh1: pJL4 for LexA-FHA (aa 1–230 of Fkh1), pJL5 for LexA-interdomain (aa 163–302), and pJL6 for LexA-forkhead (aa 231–484), respectively. LexA-fused sequences from these plasmids were integrated to the *arg5,6* locus of ECY406 (Figure 2A) to generate yeast strains YJL019, YJL020 and YJL021, respectively. For YJL094, LexA-fused sequences (LexA-FHA-R80A) from pJL8 were integrated. (B) FHA domain of Fkh1 is responsible for the regulation of donor preference. Donor preference was measured by Southern blot in panels B and C. (C) The phosphothreonine binding motif of FHA domain plays a critical role in the regulation of donor preference. XW652 and ECY406 serve as positive and negative controls, respectively.

Fkh1 contains two conserved domains: a forkhead-associated (FHA) and a forkhead DNA binding domain (Figure 3A) [22], [23]. To understand roles of different domains of Fkh1 in the regulation of donor preference, we prepared three plasmid constructs by fusing LexA of pAT4 with different regions of Fkh1: pJL4 for LexA-FHA (aa 1–230 of Fkh1), pJL5 for LexA-interdomain (aa 163–302), and pJL6 for LexA-forkhead (aa 231–484) (Figure 3A). The LexA fused sequences from these plasmids were integrated at *arg5,6* locus of ECY406 to generate strains YJL019, YJL020, and YJL021, respectively (Figure 3A). These three strains and ECY457 (Figure 2A) all have a wild-type Fkh1, which is not functional in donor preference because Fkh1 cannot bind to REΔ::LexA_BD_4. Southern blots revealed that only YJL019 could re-establish donor preference to 90%, whereas YJL020 and YJL021 failed to increase *HML* usage (Figure 3B). This result suggests that the FHA domain may play a critical role in the regulation of donor preference.

We noted that donor preference regulated by LexA-FHA_Fkh1_ (90% donor preference for YJL019; Figure 3B) was much higher than that by LexA-Fkh1 (32% donor preference for ECY457; Figure 2B). We suggest two possible explanations for this difference. First, two DNA binding domains (LexA and the forkhead DNA binding domain) are present in LexA-Fkh1, whereas only one (LexA) is present in LexA-FHA_Fkh1_. Therefore, the LexA-Fkh1 fusion protein likely binds multiple sites in yeast genome, which could mean that less fusion protein is available for regulating donor preference. In contrast, because there is only one DNA binding domain for LexA-FHA_Fkh1_, all fusion protein will be available for donor preference regulation. A second possible reason is that the FHA_Fkh1_ domain is more exposed in LexA-FHA_Fkh1_ than in LexA-Fkh1 when both fusion proteins bind to four LexA operators replacing RE. The presence of a forkhead domain in LexA-Fkh1 could interfere with regulation of the FHA_Fkh1_ domain in donor preference, whereas this kind of interference is not present in LexA-FHA_Fkh1_.

The FHA (forkhead-associated) domain is a small protein module that can preferentially bind to phosphothreonine residues on proteins [22], [24], [25]. FHA domains have been found in a wide range of proteins, such as kinases, phosphatases and transcription factors [23], [26]. To confirm that the FHA_Fkh1_ domain was responsible for increasing *HML* usage, LexA-FHA-R80A from pJL8 was integrated into the *arg5,6* locus of ECY406 to generate a strain YJL094 (Figure 3A). Preferential usage of *HML* was completely abolished using LexA-FHA-R80A (Figure 3C), which carried a non-functional FHA domain [22], [23]. Thus, the phosphothreonine-binding motif of the FHA domain plays a critical role in the regulation of donor preference.

### The FHA Domain of Fkh1 Physically Interacts with the *MAT* Region after DSB Induction

**Figure 4:**
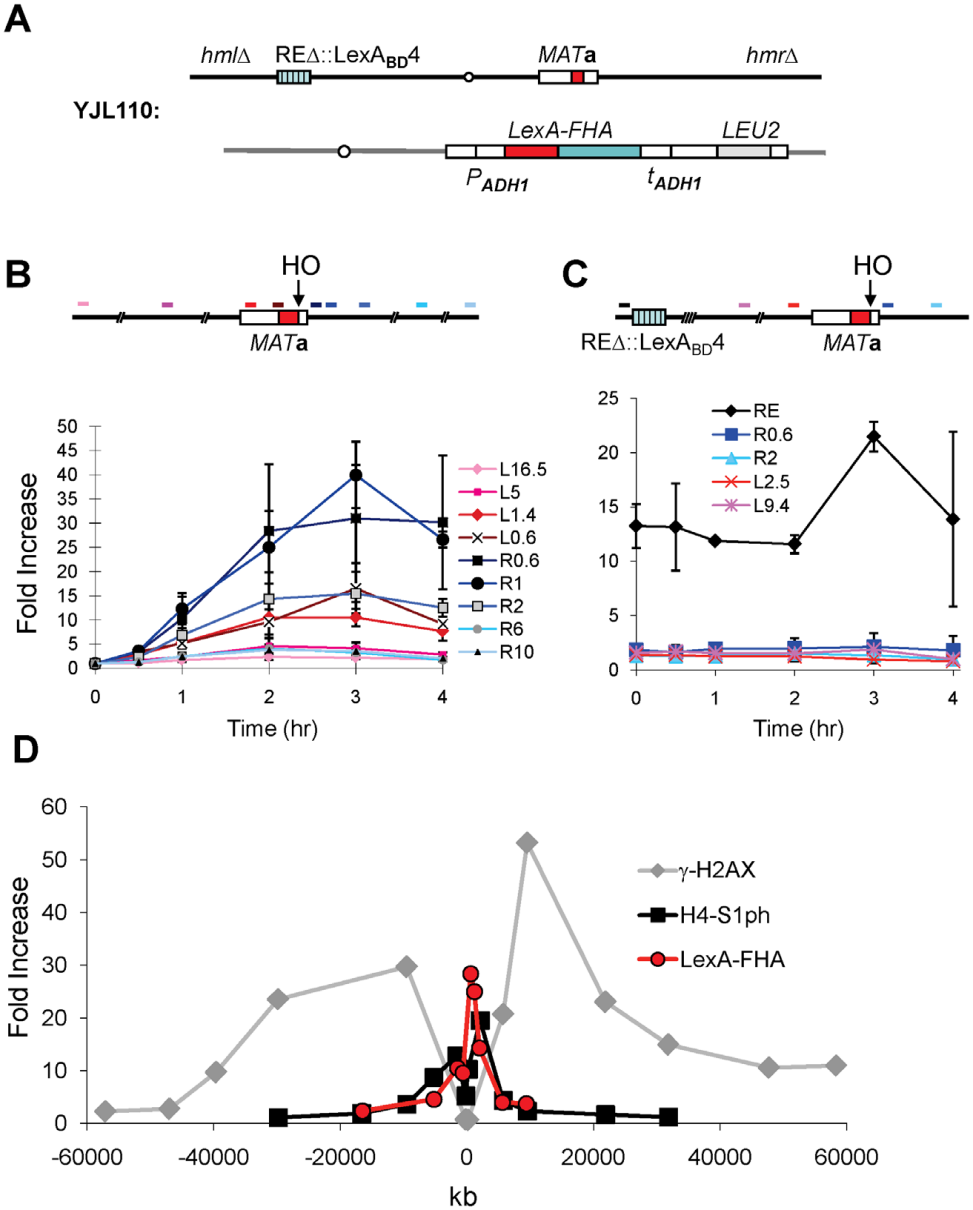
The FHA Domain of Fkh1 Physically Interacts with the *MAT* after DSB Induction. (A) Chromosome III and relevant strain genotypes for YJL110. This donorless strain is same as YJL019 (Figure 3A) except that *HML* and *HMR* are deleted. Both YJL019 and YJL110 have a wild-type Fkh1, which is not functional in donor preference because Fkh1 cannot bind to REΔ::LexA_BD_4. (B) LexA-FHA fusion protein physically interacts with the *MAT* after DSB induction. YJL110 was grown in YP-galactose and subjected to ChIP using anti-LexA antibody. The primer pair L16.5 is 16.5-kb proximal (left) from the HO site of *MAT*a, whereas R10 is located 10-kb distally (right). All other primer pairs are named and color-coded accordingly. The approximate position of each primer pair is shown in the above diagram. Immunoprecipitation (IP) signals were quantified via real-time PCR, and IP signal at each locus was normalized to that of a control locus *CEN8*. Y axis represents IP signal as fold increase relative to the IP signal at same locus before HO induction (time zero). Error bars are calculated from three repeated experiments. (C) The phosphothreonine binding motif of FHA domain is responsible for its physical interaction with the *MAT* region. Primer pairs in this panel are named similarly as in panel B, and the position of each primer pair is indicated in the above diagram. YJL094 used in the ChIP carries LexA-FHA-R80A (non-functional FHA domain) at *arg5,6* locus (Figure 3A). IP signal at each locus was normalized to that of a control locus *CEN8*. Then, these normalized IP signals (Y axis) are directly plotted against different time points following HO induction. Error bars are ranges from two repeated experiments. (D) Comparison of the distributions of γ-H2AX and histone H4-S1 phosphorylation around a DSB at *MAT* in a donorless strain JKM179 deleted for both *HML* and *HMR* [53]. ChIP values for γ-H2AX and H4-S1 phosphorylation, taken from JKM179 at 1 hr after HO induction, were normalized against input DNA, whereas LexA-FHA data, collected from YJL110 at 2 hr after induction (Figure 4B), were normalized versus *CEN8*. Y axis represents IP signal as fold increase relative to the IP signal at the same locus before HO induction (time zero).

We employed Chromatin Immunoprecipitation (ChIP) to ask if LexA-FHA_Fkh1_ could associate with the region around *MAT* before or after induction of a DSB. Using an anti-LexA antibody, we showed that LexA-fused FHA_Fkh1_ physically interacted with the *MAT* region after DSB induction in a strain lacking *HML* and *HMR* (Figure 4A), so that DSBs could not be repaired by homologous recombination. We observed a >10-fold increase in ChIP signals within about 5 kb on either side of the HO cleavage site at the *MAT*, whereas no significant signal could be detected using primer pairs that amplify regions further away from the HO site (Figure 4B). Therefore, the LexA-FHA_Fkh1_ fusion protein physically interacted with the DSB-cut *MAT* through a repair-independent mechanism, which suggests that LexA-FHA_Fkh1_ or RE can be used to stimulate recombination between any two homologous sequences in budding yeast [11].

We note that the localization of LexA-FHA_Fkh1_ binding is quite different from the roughly 50-kb phosphorylation of histone H2A-S129 (γ-H2AX) on either side of the DSB [27], [28], although it is similar to a second damage-induced modification, which is the casein kinase II-dependent phosphorylation of H4-S1 (Figure 4D) [29].

Given that RE activity was completely abolished in the strain YJL094 carrying LexA-FHA-R80A (Figure 3C), it was not unexpected that the ChIP assay showed no physical interaction between LexA-FHA-R80A and the *MAT* region after DSB induction (Figure 4C); However, the LexA-FHA-R80A fusion protein still strongly associated with REΔ::LexA_BD_4 likely due to the presence of the LexA domain (Figure 4C). These data strongly support the idea that the FHA domain of Fkh1 regulates donor preference by physically interacting with the *MAT* region during mating-type switch, and these interactions fully depend on the phosphothreonine binding motif of the FHA_Fkh1_ domain.

### RE Accelerates the Rate of DSB–Induced Ectopic Recombination for Non-*MAT* Sequences

**Figure 5:**
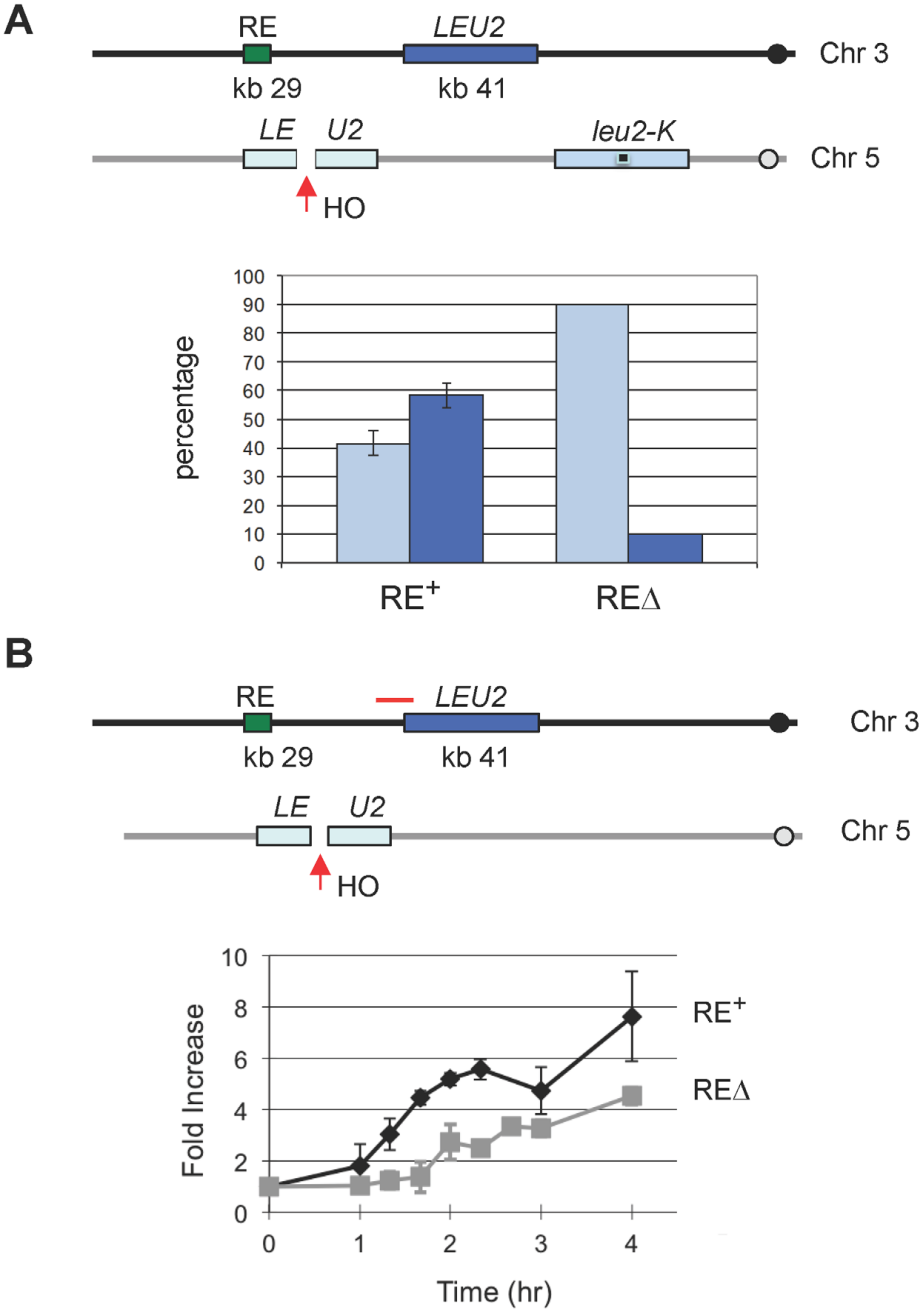
The Presence of RE Promotes DSB–Mediated Interchromosomal Gene Conversion and Accelerates Rad51 Synapse Formation. (A) The presence of RE promotes the usage of its adjacent inter-chromosomal donor for DSB repair. An HO cut site was previously introduced to the *Kpn*I site of the *LEU2* to generate *leu2::HOcs* [54]. YCSL001 (as depicted) contains the *leu2::HOcs* at the *can1* locus on chromosome V. HO-induced DSBs can be repaired via gene conversion using one of two donors: *LEU2*, inserted ∼12 kb proximal to RE on chromosome III, and *leu2-K*, lacking a *Kpn*I site, integrated as part of a Yip5 plasmid at the *ura3-52* locus. Cells were plated on YP-galactose to induce DSBs. The repaired region of each survivor was amplified by PCR, followed by *Kpn*I digestion to determine which donor was used for repair. The bar represents the percentage of repair events using either donor. Light blue bars show the use of *leu2-K* while dark blue bars indicate the use of the ectopic *LEU2* on chromosome III. YCSL003 is same as YCSL001, except that RE is deleted. For YCSL001 (RE^+^), error bars are calculated from three experiments; for YCSL003 (REΔ), values are the same for two experiments (20 colonies per experiment). (B) The presence of RE accelerates Rad51 synapse formation. In YSJ119 (as depicted), the *LEU2* on chromosome III is the only homologous donor to repair the DSB on chromosome V. YCSL014 is same as YSJ119, except that RE is deleted. Both YSJ119 (RE^+^) and YCSL014 (REΔ) were grown in galactose and subjected to ChIP with anti-Rad51 antibody. IP signal was amplified using a primer pair (YCL049p1+Leu2-91082), indicated by a red solid line, which is located at the left boundary of *LEU2* on chromosome III. IP signal was normalized to that of a control locus *CEN8*. Y axis represents IP signal as fold increase relative to the IP signal at the same locus before HO induction (time zero). Error bars indicate the range of two experiments.

Because the FHA_Fkh1_ domain regulates donor preference via a repair-independent but break-dependent mechanism, it suggests that FHA_Fkh1_ domain or RE can be used to facilitate recombination between any homologous sequences in yeast genome. Previously we showed that RE stimulated *leu2* heteroallele spontaneous recombination when one of the alleles was situated in place of *HML* [11]. In that case, the nature and position of the initiating DNA lesions were unknown. Here we integrated a *leu2::HOcs* construct at *can1* locus on chromosome V, so HO-induced DSBs can recombine with a *LEU2* locus placed near RE on chromosome III (Figure 5) [30]. In one assay, *LEU2* on chromosome III could be used as a donor to repair HO-induced DSBs on chromosome V in competition with a *leu2-K* donor inserted at *ura3*, which is 85 kb from the *leu2::HOcs* (Figure 5A). The *leu2-K* allele was created by ablating *Kpn*I site in *LEU2* [31]. As shown in Figure 5A, the proportion of repair events using the interchromosomal donor was more than 50% when RE was present but fell to less than 10% when RE was deleted. In a second assay, the *LEU2* on chromosome III was the only possible donor for DSB repair. This construct allowed us to ask whether RE stimulated recombination by facilitating the earliest step, the search for homology by Rad51 recombinase bound to the resected end of the DSB. We measured the time at which Rad51 became associated with the donor (i.e. when strand invasion had occurred) by a ChIP assay analogous to that used to assay strand invasion kinetics during *MAT* switching [32], [33]. As seen in Figure 5B, the kinetics of Rad51 association with the *LEU2* donor was significantly faster when RE was present. The presence of RE also assured that the proportion of cells that completed repair was 72% compared to 37% when RE was deleted. The percentage of completed repair was determined by comparing survival on galactose plates with that on dextrose plates where HO was not induced.

### γ-H2AX Formation at RE in *MAT*a Cells Provides Additional Evidence of Direct RE-to-*MAT* Contact

**Figure 6:**
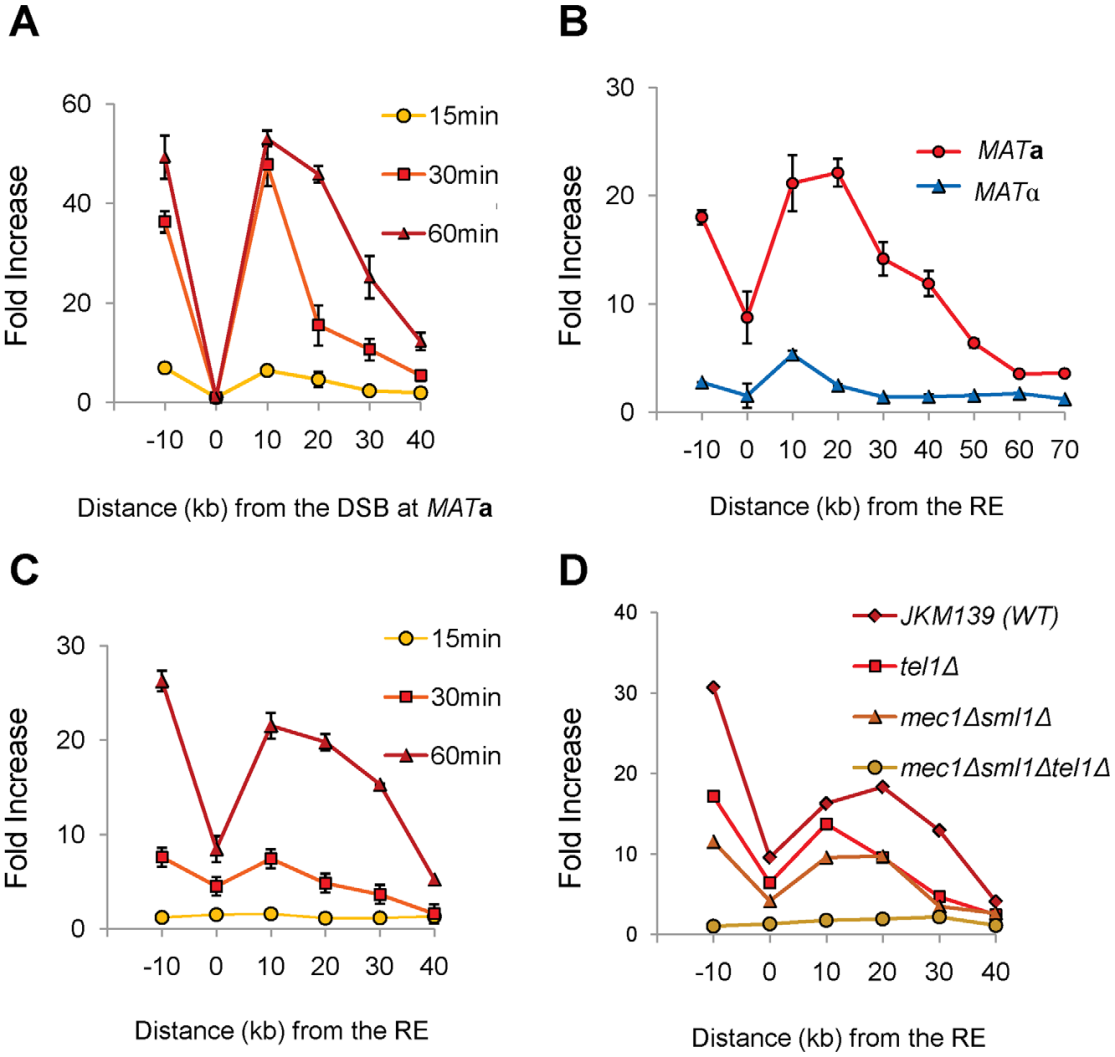
γ-H2AX Formation around *MAT* Spreads to the RE Region in *MAT*a Cells. (A) γ-H2AX formed around the *MAT* locus after DSB induction by HO. JKM139 (*MAT* **a**) lacking *HML* and *HMR* was grown in galactose and subjected to ChIP analysis with anti-γ-H2AX antibody. DNA was extracted from immune-precipitates with protein G-agarose, and IP signals around the *MAT* locus were quantified via real-time PCR using five primer pairs (−10 kb, 10 kb, 20 kb, 30 kb and 40 kb from the HO cut site). IP signal at each locus was normalized to that of a control locus *CEN8*. Y axis represents IP signal as fold increase relative to the IP signal at the same locus before HO induction (time zero). Each data point is the average of two separate experiments, with error bars representing the range of IP values. (B) γ-H2AX appeared around the RE region in *MAT* **a**, but not in *MAT*α cells. JKM139 (*MAT* **a**) and JKM179 (*MAT*α) cultured in galactose for an hour were subjected to ChIP with anti-γ-H2AX antibody as described in panel A. To test γ-H2AX PCR signals around the RE, primers pairs at various distances from RE were used. Error bars represent the range of IP values from two independent experiments. (C) Kinetics of γ-H2AX formation around RE in JKM139 (*MAT* **a**). All experimental procedures are same as described in Panel A for primer pairs amplifying regions around RE. Two independent experiments were performed and error bars represent the range of IP values. (D) The level of γ-H2AX signals around RE at 1 hr after HO induction was compared among the wild type (JKM139), *tel1Δ*, *mec1Δsml1Δ* or *mec1Δsml1Δtel1Δ* strains.

γ-H2AX rapidly forms around the site of a DSB, dependent on either Mec1 or Tel1 checkpoint protein kinase [27], [28]. If RE bound to regions around the DSB, would γ-H2AX also form around RE region? To address this question, we used ChIP with anti-γ-H2AX antibody to examine the phosphorylation of histones around RE following initiation of a DSB. γ-H2AX formed over a large domain around *MAT* following the induction of a DSB within 15–60 min (Figure 6A). Surprisingly, γ-H2AX also appeared around RE at 1 hr after HO induction in *MAT* **a** cells. As predicted, there was no similar modification around RE in *MAT*α cells, where RE is repressed (Figure 6B). Moreover, the kinetics of γ-H2AX modification around RE was slower than around *MAT*, consistent with the idea that RE first had to be recruited to the DSB before this modification could take place (Figure 6A, 6C). Finally, by using both *mec1*Δ *sml1*Δ and *tel1*Δ derivatives of JKM139, we showed that either checkpoint kinase was capable of carrying out γ-H2AX modification around RE (Figure 6D). These data provide additional supporting evidence of a direct RE-to-*MAT* contact after DSB induction and support the model that the binding of RE to *MAT* is the basis of bringing *HML* into close proximity. In addition, these data show for the first time that a region not suffering a DSB can be modified by both checkpoint kinases if this region is brought close to the DSB site.

### Are Histones the Target of FHA_Fkh1_ Domain in Donor Preference Regulation?

**Figure 7:**
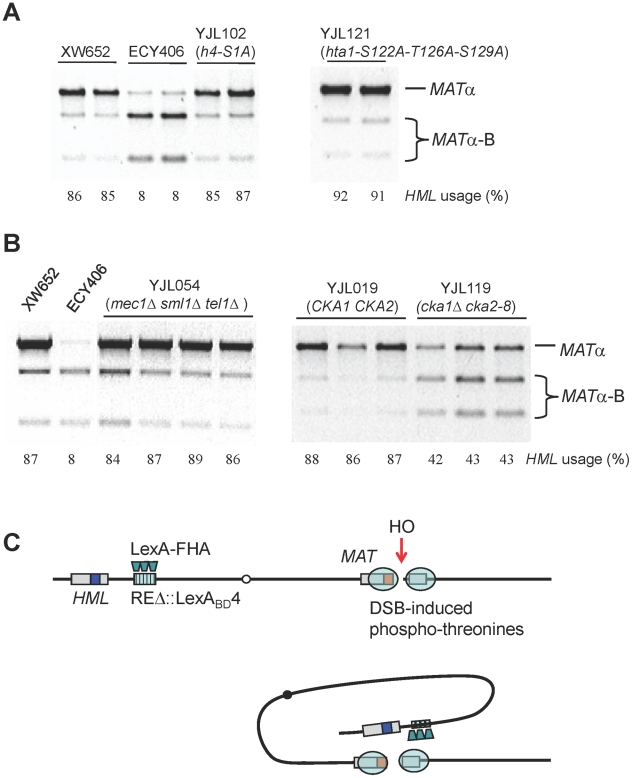
Roles of Histones and Kinases in Donor Preference and a Model for FHA-Directed Regulation. (A) The effect of H4 or H2A phosphorylation sites on donor preference. *HML* usage was not altered in strains only containing mutated *h4-S1A* or *hta1-S122A-T126A-S129A*. Donor preference was measured using a PCR-based assay (Figure 1B). (B) The effect of Mec1/Tel1 or casein kinase II on donor preference. In a triple mutant strain (YJL054), donor preference is not different from wild-type control (XW652). The *cka1::KAN*, *cka2::NAT*, pRS315-*cka2-8* (ts) are crossed into YJL019 (Figure 3A) to generate the YJL119 strain. Both strains are first cultured at 25°C for overnight and then transferred to 37°C for 3 hour incubation. Galactose induction is performed for 1 hour and stopped by the addition of 2% dextrose. (C) A model for FHA-directed regulation of donor preference. After the generation of a DSB at *MAT* **a**, we propose that a physical interaction between the FHA domain of Fkh1 and phosphothreonines of histones or bound proteins around the *MAT* will bring *HML* to the vicinity of the DSB, therefore allowing *HML* to serve as the favored template for DSB repair. The tethering of *HML* approximately 20 kb from the *MAT* can account for an almost 10-fold preference of *HML* usage over *HMR*.

Our data strongly argue that the FHA domain of Fkh1, clustered at the normal RE or REΔ::LexA_BD_4, interacts with phosphorylated residues in the region surrounding the DSB. The most obvious candidates are histones that are phosphorylated after DSB induction, including H4-S1 [29] and histone H2A-S129 (γ-H2AX). The possibility that H4-S1 could be involved was made more attractive by our finding that this modification is confined to the first 10 kb around a DSB, much more restricted than γ-H2AX (Figure 4D). We constructed a strain YJL102, carrying the h4-S1A in *HHF2* and deleted for *HHF1*; however this alteration had no effect on donor preference (Figure 7A). In addition, phosphorylation of H2A-S122, H2A-T126 and H2A-S129 have been implicated after MMS-induced DNA damage [34]. To test these H2A modifications, we constructed a strain YJL121 by deleting endogenous *HTA1-HTB1* and *HTA2-HTB2* and complementing by a plasmid carrying *hta1-S122A-T126A-S129A-HTB1*, but donor preference was not affected (Figure 7A).

We have directly tested whether post-translational modifications of the N-terminal tail of histones H3 or H4 are implicated in donor preference. In addition to H4-S1, several other sites have been reported to be phosphorylated during the cell cycle, such as H3-T3, H3-S10 and H3-S28 [35]–[37], which might also be targets for modification after a chromosome break. In particular, we constructed a strain YJK340, in which *HHF1-HHT1* was deleted with *NAT*. Then, the remaining copy of H3 gene was modified to carry a deletion of the first 32 amino acids or *HHF2* was modified to lack the first 16 amino acids of histone H4. We found that the H3 tail deletion unsilenced *HML* but not *HMR* (i.e. cells became non-mating by expressing both *HML*α and *MAT* **a**); hence we replaced the Yα sequences at *HML* with *HPH* as previously described [13]. This modification also deleted part of the HO cleavage site at *HML*, so only *MAT* **a** would be cleaved by HO. When HO was induced at *MAT*, there was no change in donor preference, as 73 of the 82 (89%) switched products were derived from *hml*::*HPH* and only 9 (11%) were *MAT*α-B, derived from *HMR*α-B. In the case of the H4 N-terminal truncation, both *HML*α and *HMR*α-B become unsilenced [38]; thus to look at donor preference, we replaced *HML*'s Yα with *HPH* and *HMR*'s Yα-B with *KAN* [13]. Among 39 colonies that switched from *MAT* **a**, 34 (87%) used *hml*::*HPH* whereas only 5 (13%) recombined with *hmr*::*KAN*. Therefore, deleting the tail of histone H3 or H4 had no effect on donor preference in *MAT*a cells.

### Fkh1-Directed Regulation Depends on Casein Kinase II, but Not on Mec1/Tel1

Although Mec1 and Tel1 can phosphorylate histone H2A in the region surrounding RE when it is brought in conjunction with *MAT*, these checkpoint kinases are not responsible for promoting *MAT* donor preference. We constructed a strain YJL054 (*mec1*Δ *tel1*Δ *sml1*Δ) derived from XW652. We noted that because Mec1 or Tel1 was required for efficient clipping of the Y**a** tail to enable the completion of switching to *MAT*α or *MAT*α-B [39], there was a strong reduction in the switching efficiency (data not shown), but the proportion of *MAT*α to *MAT*α-B was unaltered in YJL054 (Figure 7B). This conclusion that Mec1 and Tel1 are not involved in the regulation of donor preference was further supported by our data that donor preference was not altered in YJL121, in which histone H2A-S129 was mutated to alanine (Figure 7A).

Casein kinase II phosphorylates serine 1 (S1) of histone H4 after exposure to MMS- and phleomycin-induced DSBs [29] and after HO-induced DSBs (Figure 4D). Casein kinase II is required for cell cycle progression in budding yeast and essential for cell viability [40]. We constructed a strain lacking the chromosomal *CKA1* and *CKA2* genes but carrying a pRS315 plasmid with a temperature-sensitive *cka2-8* allele (Figure 7B). Cells were grown overnight at the permissive temperature of 25°C and then shifted to the restrictive temperature of 37°C. Inactivation of Cka2 leads to 43% use of *HML* (YJL119) compared to 87% donor preference in control YJL019 (Figure 7B). This result indicates that casein kinase II activity is required for Fkh1-dependent regulation of donor preference. Because the N-terminal truncation of H4 (including H4-S1) has no effect on *HML* usage, it is likely that casein kinase II phosphorylates some other targets, on a histone or another protein, which is involved in donor preference regulation. However, the fact that 43% donor preference is still significantly higher than 10% observed in RE-deleted strains (Figure 1C) suggests that multiple kinases may be involved in the regulation of donor preference.

## Discussion

We have shown that the phosphothreonine binding motif of the FHA domain of Fkh1 plays a critical role in the regulation of donor preference (Figure 3). A strong physical association between the FHA_Fkh1_ domain bound at the RE region and *MAT* is readily seen, but only after a DSB is induced. This interaction is independent of the presence of an adjacent homologous *HML* donor (Figure 4). Conversely, the region surrounding RE can be phosphorylated by Mec1 and Tel1 kinases only after DSB induction in *MAT* **a** but not in *MAT*α strains (Figure 6), again suggesting that these regions can come into physical contact when there is a DSB at *MAT* and RE is active.

RE's activity does not depend on any of the special features of *MAT* switching such as *HML* or *HMR* silencing [13] or HO cleavage [11], [15]. Consequently RE is able to improve the use of an ectopic donor in repairing a DSB on a different chromosome. Normally, a DSB will be preferentially repaired by a donor on the same chromosome in competition with an ectopic donor, but if the ectopic donor is located near RE, more than half of gene conversions use the interchromosomal donor (Figure 5A). Although our data and those from others show that *HML* is not constitutively much closer to *MAT* **a** than *HMR* is (i.e. in the absence of HO cleavage) [41]–[43], the data we present here suggest that such a reorganization will occur after a DSB is created.

Taken together, our data suggest a simple model for RE action (Figure 7C). After the induction of a DSB, casein kinase II and possibly other kinases modify some proteins bound near the DSB. These modifications, most likely phosphothreonines, are clustered near the DSB and can be bound by FHA_Fkh1_ domains tethered at RE. This binding effectively tethers *HML* within about 20 kb of the DSB whereas *HMR* remains 100 kb away. Thermodynamic considerations argue that this close proximity is sufficient to explain why *HML* should be used 90% of the time for DSB repair in *MAT* **a** cells [13]. This model also explains how RE can act over a long region of the left arm of chromosome III [8], although with diminishing effect [12], by this tethering mechanism.

The model we propose argues that RE should be portable and able to stimulate the use of any homologous donor in a DSB repair mechanism. Our previous work has shown that RE is portable, as it is able to activate *HML* use when both are inserted on chromosome V [12]. Moreover, if a copy of RE is inserted near *HMR* in a *MAT* **a** strain that also has RE near *HML*, then *HMR* usage is increased to about 50% (E.C., S.-Y. Tay and J.E.H., unpublished). The ectopic recombination experiment presented here shows that RE can act efficiently on non-*MAT* sequences for DSB repair (Figure 5A).

We note that we have previously shown that RE could stimulate spontaneous recombination between *leu2* heteroalleles when one of them was located close to the RE [11], [12]. The results we report here suggest that a large proportion of spontaneous recombination events may be triggered by DSBs or that the same phosphorylated protein attracting the attention of RE during DSB repair also accumulates at the lesions that stimulate spontaneous recombination.

At present, we have not yet identified the phosphothreonine target for the FHA domain of Fkh1. We have ruled out a number of candidates, including γ-H2AX, N-terminal tails of histones H3 and H4, as well as Mre11 and Sae2, two proteins involved in DSB end-binding and initiating 5′ to 3′ resection (C.-S. L., J.E.H., unpublished observations). Studies using peptide libraries and immunoprecipitation of the FHA_Fkh1_ domain after DSB induction are underway.

Aparicio group has recently made the intriguing finding that Fkh1 and Fkh2 proteins play a key role in the activation and clustering of early origins of replication in budding yeast [44]. This regulation involves a cis-acting association of these two forkhead proteins with proteins at origins. It will be interesting to ask if the FHA domain of Fkh1 plays an important role in this regulation.

Another important finding emerging from our work is that two DNA damage checkpoint kinases, Mec1/ATR and Tel1/ATM, can act to phosphorylate distant DNA sequences when they are tethered in the vicinity of the DSB. As shown in Figure 6, the γ-H2AX modification spreads around the RE region, but with significantly delayed kinetics compared with the modification around *MAT*, consistent with the idea that RE has to first recognize and bind to phosphorylated residues in the vicinity of the DSB at *MAT*. How these checkpoint kinases act on their target sequences is not yet firmly established. Mammalian ATM has been shown to be activated by intermolecular autophosphorylation and dimer exchange, which would suggest that activated ATM would initially form a “cloud” of activated kinases around the site where the kinases were associated with the DSB ends [45], [46]. In the case of Tel1/ATM, the association with the DSB is via its association with the MRX/MRN proteins [47], [48]; in the case of Mec1/ATR, by its association its partner protein Ddc2/ATRIP with RPA bound to ssDNA at the resected DSB end [49], [50]. In budding yeast, the spreading of γ-H2AX from the DSB site is consistent with that the tethered kinases interact with phosphorylating histones on the adjacent chromosomal segment in a manner, which is similar to the contact of chromosomal regions as measured in chromosome conformation capture experiments [51]. Spreading of γ-H2AX further along the chromosome occurs more slowly and apparently depends on the continuing 5′ to 3′ resection of the DSB ends, generating ssDNA, as it depends only on Mec1 [27], [28]. Here we show that histones in another distant chromosomal region, brought into proximity with the DSB by RE, can also be efficiently phosphorylated – and by both Mec1 and Tel1. This result is different from the slow addition of γ-H2AX to regions further from the DSB, which depends on continuing 5′ to 3′ resection of the DSB ends and can only be performed by Mec1 [27], [28]. We have also observed γ-H2AX spreading onto a different chromosome during the ectopic recombinational repair of a DSB, when these two regions are brought together by Rad51-mediated strand invasion (K.L. and J.E.H., unpublished observations).

## Materials and Methods

### Yeast Strains

All strains except when noted were derived from strain XW652 (*ho ade3::GAL::HO HML*α *RE MAT* **a** *HMR*α-B *ura3-52 lys5 leu2-3,112 trp1::hisG*) carrying a galactose-inducible HO endonuclease integrated at the *ADE3* locus [11]. Strains are pre-cultured in YP-lactate medium until cell density reaches about 5∼8×10^6^ per ml. Galactose induction is performed for 1 hour and stopped by the addition of 2% dextrose.

Construction of ECY406 (Figure 2A): Four LexA operators are amplified from pSH18-34 [52] using primers BglIILexAU (5′-cga cga gat cta tac ata tcc ata tct aat ctt acc-3′) and BglIILexAL (5′-gct gca gat ctc taa tcg cat tat cat ccc tcg a-3′). Then PCR products were digested with *Bgl*II and subcloned into the *BamH*I site of pKS58 to generate pEC15. The SphI-digested pEC15 (marked with “*LEU2*”) was transformed into XW676 (*ho ade3::GAL::HO HMLα REΔ::URA3 MAT* **a** *HMRα-*B *ade1 leu2 trp1 ura3-52*) to replace *REΔ::URA3* with four LexA operators to generate a strain ECY405. Then, *REΔ::LexA_BD_4-LEU2* from ECY405 was replaced with *REΔ::LexA_BD_4-KAN* to generate a strain ECY406 (Figure 2A) via transformation using PCR fragments amplified from pJH1894 with primers leu2KanU (5′-gag aac ttc tag tat atc cac ata cct aat att att gcc tta tta aaa atc agc tga agc ttc gta cgc-3′) and leu2KanL (5′-tac gtc gta agg ccg ttt ctg aca gag taa aat tct tga ggg aac ttt cag cat agg cca cta gtg gat ctg-3′).

ECY457 (Figure 2A) is constructed by transforming ECY406 with PCR fragment *arg5,6::LexA-Fkh1* obtained with primers pAT4UII (5′-atg cca tct gct agc tta ctc gtc tcg aca aag aga ctt aac gct tcc aaa ttc cat ttt gta att tcg tgt cg-3′) and pAT4LII (5′-tca gac acc aat aat ttt att ttc agg gat acc agc ata ctc tcc ata aca agg gaa caa aag ctg gag c-3′) on the plasmid pEC16. Using a similar strategy, PCR products *arg5,6::LexA-FHA* (from pJL4), *arg5,6::LexA-interdomain* (from pJL5), *arg5,6::LexA-forkhead* (from pJL6) and *arg5,6::LexA-FHA-R80A* (from pJL8) are transformed into ECY406 to generate YJL019, YJL020, YJL021 and YJL094, respectively (Figure 3A).

YJL084 was made by transforming YJL019 (Figure 3B) with *BamH*I digested pJH1250 to delete *HML* using the *URA3* marker. YJL110 (Figure 4A) is made by transforming YJL084 with BsaI-digested pJH2039 to delete *HMR* using the *NAT* marker.

Yeast strains with H3 or H4 N-terminal truncation were constructed by sequential transformations of JKM139 [32]. The *HHF1-HHT1* allele of JKM139 was first knocked out by NAT-MX cassette to generate a strain YJK340 (*ho ade3::GAL::HO hmlΔ::ADE1 RE MAT* **a** *hmrΔ::ADE1 ade1 leu2-3-112 lys5 ura3-52 trp1::hisG hhf1-hht1Δ::NAT*). Then, YJK340 was transformed with linearized plasmid carrying *hht2-hhf2* mutant alleles linked to *URA3* marker to replace endogenous *HHT2-HHF2* allele. *HHT2* was modified to lack the first 32 amino acids of histone H3 or *HHF2* was modified to lack the first 16 amino acids of histone H4. To prepare for mating-type switching assay, *HMLα* and *HMRα-*B from XW652 were crossed into a yeast strain with H3 or H4 N-terminal truncation.

### Measure Donor Preference via Southern Blot or a PCR-Based Assay

All strains except when noted in this study are derived from XW652 (*ho ade3::GAL::HO HML*α *RE MAT* **a** *HMR*α-B *ura3-52 lys5 leu2-3,112 trp1::hisG*) [11]. The C→A change at position 658 of *Yα* creates a *BamH*I restriction site (*HMRα*-B), which is absent in *HML*α [8]. Donor preference (*HML* usage) is calculated using the formula (*MAT*α/(*MAT*α+*MATα*-B) for all XW652 derived strains (Figure 1A). The measurement of donor preference via Southern blot was described previously [8]. Southern signals were quantified using ImageQuant V1.2 (Molecular Dynamics).

Because there is only 1-bp difference between two repaired products (*MATα* and *MATα-*B), we have developed a PCR-based assay to measure donor preference. The presumption is that PCR amplification efficiency is almost identical for *MATα* and *MATα-*B because there is only 1-bp difference [8]. Around 10 ng of genomic DNA isolated from galactose-induced colonies will be used for PCR amplification. Two primers Yalpha105F (5′-gcc cac ttc taa gct gat ttc aat ctc tcc-3′) and MATdist-4R (5′-cct gtt ctt agc ttg tac cag agg-3′) can only amplify *MAT*α or *MATα-*B, but not *MAT* **a**, *HML*α or *HMRα*-B due to sequence specificities of these two primers (Figure 1B). Although amplified PCR products are the mixture of *MAT*α-B and *MAT*α, only one 1470-bp band can be visualized on DNA agarose gel prior to digestion. PCR products are then purified and subsequently digested with *BamH*I. The digested PCR products will be checked on DNA agarose gel. *MAT*α product will remain as the 1470-bp band, whereas *MAT*α-B product is digested into two smaller bands with different sizes (550-bp and 920-bp) (Figure 1C). Donor preference is determined by comparing intensities of *MAT*α and *MAT*α-B after the agarose gel is stained with ethidium bromide.

### Plasmid Constructs

To study if Fkh1 can regulate donor preference in our LexA system, we construct a LexA-Fkh1 fusion plasmid (pEC16) carrying the coding sequence of Fkh1. Fkh1 coding sequence is PCR amplified from XW652 genomic DNA using primers XmaIFkh1U (5′-tcg cga ccc ggg gat ccg tat gtc tgt tac cag tag gg-3′) and PstIFkh1L (5′-gca cga cct gca gtc aac tca gag agg aat tgt tca cg-3′). The amplified PCR product is digested with XmaI and PstI and then subcloned into a pre-digested pAT4 [21] to generate the plasmid pEC16.

To address different roles of Fkh1 domains in the regulation of donor preference, three regions of Fkh1 are subcloned into pAT4 (Figure 3A). The FHA domain of Fkh1 is amplified via PCR using primers XmaIFkh1U and PstIFkh1-690L (5′-gca cga cct gca gta ggt ggt cca gct gtt gta atc g-3′). The interdomain region is amplified using primers XmaIFkh1-487U (5′-tcg cga ccc ggg gat cgg tgt gca aat gat ctt tat at-3′) and PstIFkh1-906L (5′-gca cga cct gca gga tat atc tgt ttt cat cca gc-3′). The forkhead domain is amplified using primers XmaIFkh1-691U (5′-tcg cga ccc ggg gat cca cac ccc att atc gtc atc at-3′) and PstIFkh1L. These PCR products are then digested with XmaI and PstI, and subcloned into a pre-digested pAT4, to generate three fusion plasmids pJL4, pJL5 and pJL6, respectively.

### Site-Directed Mutagenesis of pJL4

Quickchange Multi Site-Directed Mutagenesis Kit (Catalog # 200515, Stratagene, La Jolla, CA) was used to mutate the FHA domain of pJL4. Two primers Fkh1-Arg80 (5′-tta gaa gtt acc att ggt gcg aac aca gac agc ttg aac-3′) and pAT4-940R (5′-ctt tgc cag aca aga aca ccg cat-3′) were used to synthesize mutant strand from pJL4. Fkh1-Arg80 shares two-base mismatches with Fkh1 and pAT4-940R perfectly matches pJL4. The mutated plasmid pJL8 (pLexA-FHA-R80A) was confirmed by direct sequencing.

### Chromatin Immunoprecipitation (ChIP)

Procedures for ChIP analysis were described previously [15]. Rabbit anti-LexA polyclonal antibody (Catalog no. 39184) used in ChIP assay is purchased from “Active Motif” company (Carlsbad, CA). LexA ChIP signals are quantified with real-time PCR using a Chromo 4 machine from MJ Research. The linearity of PCR signals is monitored with r-square value of a calibration curve, which is prepared using a series of dilutions of the 0 hr input sample. IP signal is determined by comparing to the calibration curve, and then normalized to the IP signal of a control locus *CEN8*. PCR primer sequences around the *MAT*, RE and the ectopic *leu2::HOcs* are available on request.
